# Factors that predict lymph node status in clinical stage T1aN0M0 lung adenocarcinomas

**DOI:** 10.1186/1477-7819-12-42

**Published:** 2014-02-21

**Authors:** Bo Ye, Ming Cheng, Xiao-Xiao Ge, Jun-Feng Geng, Wang Li, Jian Feng, Ding-Zhong Hu, Heng Zhao

**Affiliations:** 1 Department of Thoracic Surgery, Shanghai Chest Hospital, Shanghai Jiaotong University, Huaihaixi Road 241, Shanghai 200030, China; 2 Med-X Renji Clinical Stem Cell Research Center, Renji Hospital, Shanghai Jiao Tong University School of Medicine, Shanghai China

**Keywords:** Lymph node, Lung adenocarcinomas, Stage small non-small cell lung cancer

## Abstract

**Background:**

To identify patients in whom systematic lymph node dissection would be suitable, preoperative diagnosis of the biological invasiveness of lung adenocarcinomas through the classification of these T1aN0M0 lung adenocarcinomas into several subgroups may be warranted. In this retrospective study, we sought to determine predictive factors of lymph node status in clinical stage T1aN0M0 lung adenocarcinomas.

**Methods:**

We retrospectively reviewed the records of 273 consecutive patients undergone surgical resection of clinical stage T1aN0M0 lung adenocarcinomas at Shanghai Chest Hospital, from January 2011 to December 2012. Preoperative computed tomography findings of all 273 patients were reviewed and their tumors categorized as pure GGO, GGO with minimal solid components (<5 mm), part-solid (solid parts >5 mm), or purely solid. Relevant clinicopathologic features were investigated to identify predictors of hilar or mediastinal lymph node metastasis using univariate or multiple variable analysis.

**Results:**

Among the 273 eligible clinical stage T1aN0M0 lung adenocarcinomas examined on thin-section CT, 103 (37.7%) were pure GGO, 118 (43.2%) GGO with minimal solid components, 13 (4.8%) part-solid (solid parts >5 mm, five GGO predominant and eight solid predominant), and 39 (14.3%) pure solid. There were 18 (6.6%) patients with lymph node metastasis. Incidence of N1 and N2 nodal involvement was 11 (6.6%) and seven (2.6%) patients, respectively. All patients with pure GGO and GGO with minimal solid components (<5 mm) tumors were pathologically staged N0. Multivariate analyses showed that the following factors significantly predicted lymph node metastasis for T1a lung adenocarcinomas: symptoms at presentation, GGO status, and abnormal carcinoembryonic antigen (CEA) titer. Multivariate analyses also showed that the following factors significantly predicted lymph node metastasis for pure solid tumors: air bronchogram sign, tumor size, symptoms at presentation, and abnormal CEA titer.

**Conclusions:**

The patients of clinical stage T1aN0M0 lung adenocarcinomas with pure GGO and GGO with minimal solid components tumors were pathologically staged N0 and systematic lymph node dissection should be avoided. But systematic lymph node dissection should be performed for pure solid tumors or part-solid, especially in patients with CEA greater than 5 ng/mL or symptoms at presentation, because of the high possibility of lymph node involvement.

## Background

The introduction of computed tomography (CT) to screen for lung cancer has made it possible to detect early-stage lung adenocarcinomas [1], thus improving patient survival [2]. Based on a randomized trial performed in 1995, the current standard surgical treatment for clinical stage IA non-small cell lung cancer (NSCLC) is lobectomy with systematic lymph node dissection [3]. Since 1995, several authors have reported that lung adenocarcinomas with large areas of ground glass opacity (GGO) have good prognosis: in most cases their pathologic features indicate they are minimally invasive [4-8], whereas mixed GGOs or pure solid tumors are more prone to lymph node metastasis.

Since there is no well-established means of predicting lymph node metastasis, systematic lymph node dissection for clinical stage T1aN0M0 lung adenocarcinomas is controversial; no consensus has yet been reached. Classifying clinical stage T1aN0M0 lung adenocarcinomas into subgroups according to their likely invasiveness and thus identifying patients in whom systematic lymph node dissection is more strongly indicated would be helpful. In this retrospective study, we sought to identify factors that predict lymph node status of clinical stage T1aN0M0 lung adenocarcinomas.

## Methods

We retrospectively reviewed the records of 5,312 consecutive patients of NSCLCs who underwent surgical resection at the Department of Thoracic Surgery, Shanghai Chest Hospital, Shanghai Jiaotong University, Shanghai, China, from January 2011 to December 2012. This study was conducted in accordance with the Declaration of Helsinki. The Shanghai Jiaotong University institutional review board approved this study. Because this was a retrospective study, informed consent was waived.

Preoperative contrast-enhanced chest CT scans are routinely performed at our hospital, even in patients who have had CT scans in other hospitals. For nodules less than 2 cm in diameter, high resolution thin-section CT with 2 mm collimation is usually performed. The lung was photographed with a window level of -500 to -700 Hounsfield units and a window width of 1,000 to 2,000 Hounsfield units as a 'lung window’. Other routine preoperative tests included cardiopulmonary tests, brain magnetic resonance imaging or brain CT, bone scanning, and abdominal CT orabdominal ultrasonography. One of the authors (JFG), who is a radiologist, was blinded to the eventual lymph node status when reviewing the scans.

According to the findings on thin-section CT, the tumors were allocated to one of three groups: pure GGO, mixed GGO, and pure solid tumors. The mixed GGO was subdivided into GGO with minimal solid components (<5 mm) and part-solid (solid parts >5 mm, subdivided into GGO predominant and solid predominant). A GGO was defined as an area of a slight homogeneous increase in density that did not obscure the underlying vascular markings, a mixed GGO tumor was defined as one with both GGO and solid components, and a pure solid tumor was defined as having only solid components without any ground glass appearance. There were 273 (5.1%) patients with clinical stage T1aN0M0 lung adenocarcinomas with pure GGO, mixed GGO, or solid appearance on CT.

A lymph node was considered positive if its short axis exceeded 1 cm on chest CT images. The inclusion criteria were as follows: 1) a single clinical stage T1aN0M0 lung adenocarcinoma and no other nodules in either lung; 2) the patient had undergone systematic lymph node dissection (removal of all lymphatic tissues within defined anatomic landmarks of stations 2 to 4 and 7 to 12 on the right side, and stations 4 to 12 on the left side according to the classification of the American Thoracic Society); and 3) the patient had undergone pulmonary segmentectomies or pulmonary lobectomy. The exclusion criteria were as follows: 1) evidence of positive lymph nodes or distant metastasis on CT scans; 2) having received neoadjuvant chemotherapy or radiotherapy; and 3) history of malignant tumors.

The medical record of each patient was reviewed with regard to age, gender, smoking history, symptom (including cough, bloody phlegm, low-grade fever, chest pain, and chest distress), tumor diameter, GGO status (GGO, mixed GGO, and pure solid), pleural involvement, presence of an air bronchogram sign on radiography, and serum carcinoembryonic antigen (CEA) level (ng/mL). The relationships between these factors and postoperative nodal status were investigated to identify significant predictors in clinical stage T1aN0M0 lung adenocarcinomas. To compare pairs of factors, χ^2^ and Fisher’s exact tests were used. Univariate and multivariate analyses were performed to identify the factors that predicted nodal involvement. Multiple variable analysis was performed by logistic regression analysis using IBM SPSS Statistics, version 20 (IBM Corporation, Armonk, NY, USA). Forward and backward stepwise procedures were used to determine the combinations of factors that were essential for predicting the prognosis. *P* <0.05 was considered to be significant.

## Results

Among the 273 eligible lung cancers, 103 (37.7%) presented as pure GGO, 118 (43.2%) GGO with minimal solid components, 13 (4.8%) part-solid (solid parts >5 mm, five GGO predominant and eight solid predominant), and 39 (14.3%) pure solid on thin-section CT. Overall, 128 of the patients were men and 145 were women. Their average age was 62.4 years (range: 32 to 84 years). Incidence of N1 and N2 nodal involvement was 11 (4%) and seven (2.6%) patients, respectively. Of the seven pN2 patients, six (85.7%) also had involvement in N1-level lymph nodes and one (14.3%) had nodal skip metastasis.

According to univariate analysis of factors possibly associated with lymph node metastasis, age at diagnosis, gender, smoking history, tumor size, or air bronchogram sign were not associated (Table 1). All patients with pure GGO tumors and GGO with minimal solid components (<5 mm) were pathologically staged as N0. Although pathologic lymph node involvement was found in 16 (41%) patients with pure solid tumors (ten patients with involvement in N1 stations and six with involvement in N2 stations, respectively), such involvement was seen in only two (1.5%) of the patients with part-solid (solid parts >5 mm, solid predominant) tumors (one patient with involvement in N1 stations and one with involvement in N2 stations, respectively). The relationships between GGO status, the mode of surgical resection, and the pathologic aspects are presented in Table 2. Standard lobectomy was performed in 197 (72.2%) patients (nine patients with involvement in N1 stations and three with involvement in N2 stations, respectively) and segmentectomy in 76 (27.8%) patients (four patients with involvement in N1 stations and two with involvement in N2 stations, respectively). No lymph node metastases were found in atypical adenomatous hyperplasia (AAH) (20/273, 7.3%), adenocarcinoma in situ (AIS) (78/273, 28.6%), and minimally invasive adenocarcinoma (MIA)(133/273, 76.9%). Correlation of radiologic classification with pathological type of tumor revealed that pure GGOs are almost all AAH or AIS (20 AAH (19.4%); 78 AIS (75.7%)). Almost all patients with mixed GGOs were found to have MIA on pathological examination (127 patients, 96.9%), whereas almost all patients with pure solid tumors had invasive adenocarcinoma (38 patients, 97.4%). One MIA case showing a pure solid nodule was a case of mucinous MIA.

**Table 1:** Prognostic factors and results of univariate analysis for predictors of pathologic nodal involvement in 273 patients with clinical stage T1aN0M0 lung adenocarcinomas

**Variable**	**Total (n = 273)**	**pN0 (n = 255)**	**pN1(n = 11)**	**pN2(n = 7)**	***P *** **value**^**a**^
Age (years)					0.643
≤59	112	103	6	3	
>59	161	152	5	4	
Gender					0.857
Female	145	136	5	4	
Male	128	119	6	3	
Smoking history					0.916
Never	164	154	6	4	
Current or former	109	101	5	3	
Symptoms					0.001
Absent	228	218	5	5	
Present	45	37	6	2	
Tumor size (cm)					0.854
≤1.0	59	56	2	1	
>1.0, ≤2.0	214	199	9	6	
GGO status					<0.001
Pure GGO	103	103	0	0	
GGO with minimal solid components (<5 mm)	118	118	0	0	
Part-solid (solid parts >5 mm)					
GGO predominant	5	5	0	0	
Solid predominant	8	6	1	1	
Pure solid	39	23	10	6	
Pleural involvement					0.024
Negative	183	176	5	2	
Positive	90	79	6	5	
Air bronchogram sign					0.546
Absence	157	147	5	5	
Presence	116	108	6	2	
CEA (ng/mL)					<0.001
≤5	209	204	3	2	
>5	64	51	8	5	
Pathologic type					<0.001
Atypical adenomatous hyperplasia (AAH)	20	20	0	0	
Adenocarcinoma in situ (AIS)	78	78	0	0	
Minimally invasive adenocarcinoma (MIA)	133	133	0	0	
Invasive adenocarcinoma	42	24	10	8	

^a^*P* value in χ^2^ test or Fisher’s exact tests. CEA, carcinoembryonic antigen; GGO, ground glass opacity.

**Table 2:** **Relationships between GGO status**, **surgical interventions**, **and pathologic aspects**

**Surgico-pathological features**	**Pure GGO (n = 103)**	**Mixed GGO (n = 131)**	**Pure solid (n = 39)**	***P *** **value**
Operative mode				<0.001
Segmentectomy	40	34	2	
Lobectomy	63	97	37	
Nodal involvement				<0.001
N0	103	129	23	
N1	0	1	10	
N2	0	1	6	
Pathologic type				<0.001
Atypical adenomatous hyperplasia (AAH)	20	0	0	
Adenocarcinoma in situ (AIS)	78	0	0	
Minimally invasive adenocarcinoma (MIA)	5	127	1	
Invasive adenocarcinoma	0	4	38	

GGO, ground glass opacity.

Multiple variable analysis of age at diagnosis, gender, symptom(s) at presentation, smoking, tumor diameter, GGO status (GGO, mixed GGO, or pure solid tumor), pleural involvement, presence of air bronchogram sign on radiography, serum CEA concentration (ng/mL), and pathologic type showed that the following factors significantly predicted lymph node metastasis: GGO status, symptoms at presentation, and abnormal CEA titer (Table 3).

**Table 3:** Results of a multiple variable analysis for predictors of nodal involvement in clinical stage T1aN0M0 lung adenocarcinomas

**Variable**	**OR**	**95% CI for HR**	***P *** **value**
GGO status	44.212	7.901 to 247.39	<0.001
Symptoms	6.501	1.179 to 35.84	0.032
CEA	7.854	1.486 to 41.524	0.015

CEA, carcinoembryonic antigen; CI, confidence interval; GGO, ground glass opacity; HR, Hazard Ratio.

We also undertook a multiple variable analysis for clinical stage T1a pure solid tumor, including age at diagnosis, gender, symptom at presentation, smoking history, tumor diameter, pleural involvement, presence of an air bronchogram sign on radiography, serum carcinoembryonic antigen (CEA) level (ng/mL), and pathologic type (Table 4). Multivariate analyses showed that the following factors significantly predicted lymph node metastasis: air bronchogram sign, tumor size, symptoms at presentation, and abnormal CEA titer (Table 4).

**Table 4:** Results of a multiple variable analysis for predictors of nodal involvement in clinical stage T1a pure solid tumor

**Variable**	**OR**	**95% CI for HR**	***P *** **value**
Tumor size (cm)	2.01	1 to 3.98	0.021
Air bronchogram sign	4.04	2.001 to 5.99	0.035
Symptoms	70.89	1.887 to 2.663	0.02
CEA	4.467	1.514 to 8.498	0.013

CEA, carcinoembryonic antigen; CI, confidence interval; HR, Hazard Ratio.

## Discussion

Although the International Association for the Study of Lung Cancer’s 2009 guidelines suggested that clinical stage IA lung cancers (such as NSCLC) are generally less aggressive [9], this might not be true of clinical stage T1a lung adenocarcinomas. Their 2011 classification of lung adenocarcinoma is based on a multidisciplinary approach to diagnosing lung adenocarcinoma that incorporates clinical, molecular, radiologic, and surgical aspects [10], and supports the notion of arranging T1a lung adenocarcinomas into several subgroups.

Systematic lymph node dissection might be avoided in selected patients if we could reliably identify factors associated with pN0 status [11]. Although many scientific studies have been performed to identify candidates for whom systematic mediastinal lymph node dissection might be dispensable [5,8,12], our study was different from previous studies in several ways. First, to our knowledge, this study assessed the largest selection of possible predictive factors for lymph node status in these cancers. Second, we included histologic subtypes as potential predictors of lymph node metastasis in line with the new classification in 2011 [10]. Third, this study made clinical stage T1a lung adenocarcinomas a separate object of study. Fourth, we used logistic regression to create a probability formula for lymph node metastasis.

Some previous studies have suggested that T1a lung cancers are not associated with mediastinal lymph node metastasis [12,13]. However, in our study we found 18 cases (6.6%) of lymph node metastasis in the patents of T1a lung adenocarcinomas; we therefore believe that these patients require systematic lymph node dissection.

Our study also indicated that clinical stage T1a lung adenocarcinomas have different invasive patterns. Hattori *et al*. [14] believe that tumor diameter, pure solid, pleural involvement, presence of an air bronchogram sign on radiography, high CEA level (>5 ng/mL), and high maximum standardized uptake value (SUVmax) on positron emission tomography (PET) (>5) are important predictors of lymph node metastasis for clinical stage IA lung adenocarcinomas.

Our univariate analysis of possible factors identified symptoms at presentation, GGO status, pleural involvement, serum CEA level (ng/mL), and pathologic type as predictors of nodal involvement in clinical stage T1a lung adenocarcinomas, whereas multiple variable analysis identified symptoms at presentation, GGO status, and abnormal CEA titer (>5 ng/mL) as important predictors of lymph node metastasis. Since GGO status is a more accurate predictor of lymph node metastasis than relative tumor diameter, we suggest that tumor size alone should not be used to rule out complete mediastinal lymph node dissection.

In addition, our study found no lymph node metastasis in 103 patients with GGO (including 20 with AAH (20/103, 19.4%), 78 with AIS (78/103, 75.7%), and five with MIA (5/103, 4.9%)). Among 131 patients of mixed GGO (including 127 with MIA (127/131, 96.9%) and four with invasive adenocarcinoma (4/131, 3.1%)), a total of two cases (one solid predominant and one invasive adenocarcinoma) had lymph node metastasis. Among 39 patients with pure solid performance (including 38 with invasive adenocarcinoma (38/39, 97.4%) and one with MIA (2.6%)), a total of 16 cases (16/39, 41%) had incidence of lymph node metastasis. Therefore, we believe that all patients with part-solid (solid parts >5 mm, GGO predominant and solid predominant) or pure solid tumors should undergo systematic lymph node dissection. Hattori *et al*. [14] also found that all patients with pure GGO tumors were pathologically staged N0 and there is a threshold at which part-solid, part-non-solid nodules essentially behave the same as solid nodules with the risk of nodal involvement. Our study also confirmed this.

Russell *et al*. [15] investigated the relationship between subtypes of lung adenocarcinoma and clinical characteristics in Australian patients with stages I to III and revealed that none of the AIS, MIA, lepidic-predominant adenocarcinoma (LPA), or invasive mucinous adenocarcinoma (IMA) cases had lymph node metastases; their findings concur with our results. We found no lymph node metastases among patients with AAH, AIS, and MIA, which are minimally invasive. This implies that histologic classification could help avoid systematic lymph node dissection in a large proportion of patients with clinical stage IA lung adenocarcinomas.

Based on our results, T1a lung adenocarcinomas of pure solid appearance with an invasive nature, that is, a preoperative solid appearance on thin-section CT, showed an incidence of lymph node metastasis of more than 40%. Multivariate analyses showed that the following factors significantly predicted lymph node metastasis: air bronchogram sign, tumor size, symptoms at presentation, and abnormal CEA titer. Therefore, we believe that the patients of pure solid appearance must undergo the systematic lymph node dissection.

However, our study is retrospective in essence and was limited by the lack of uniform use of PET scanning. In addition, the accuracy of GGO status to classify lung adenocarcinoma subtypes still needs to be confirmed. Therefore, a randomized trial should be performed to validate the implications of our findings.

## Conclusion

In conclusion, all patients with pure GGO tumors were pathologically staged N0 and systematic lymph node dissection should be avoided. But systematic lymph node dissection should be performed for pure solid tumors or part-solid, especially in patients with CEA greater than 5 ng/mL or symptoms at presentation, because of the high possibility of lymph node involvement. With regard to the efficacy of limited surgical resection for clinical stage T1a lung adenocarcinomas, any final conclusions should be based on the results of phase III trials conducted by JCOG0802 [16] and CALGB-140503 [17].

## Abbreviations

AAH: Atypical adenomatous hyperplasia; AIS: Adenocarcinoma in situ; CEA: Carcinoembryonic antigen; CT: Computed tomography; GGO: Ground grass opacity; IMA: Invasive mucinous adenocarcinoma; LPA: Lepidic-predominant adenocarcinoma; MIA: Minimally invasive adenocarcinoma; NSCLC: Non-small cell lung cancer; PET: Positron emission tomography; SUVmax: Maximum standardized uptake value.

## Competing interests

The authors declare that they have no conflicts of interest. The authors had full control of the design of the study, methods used, analysis of data, and content of the written report.
